# Pathogen-Specific Actinium-225 and Lutetium-177 Labeled Antibodies for Treatment of Biofilm-Associated Implant Infections: *Initial In Vivo Proof-of-Concept*

**DOI:** 10.3390/antibiotics14121283

**Published:** 2025-12-18

**Authors:** F. Ruben H. A. Nurmohamed, Kevin J. H. Allen, Mackenzie E. Malo, Connor Frank, J. Fred F. Hooning van Duvenbode, Berend van der Wildt, Alex J. Poot, Marnix G. E. H. Lam, Jos A. G. van Strijp, Peter G. J. Nikkels, H. Charles Vogely, Harrie Weinans, Ekaterina Dadachova, Bart C. H. van der Wal

**Affiliations:** 1 College of Pharmacy and Nutrition, University of Saskatchewan, 107 Wiggins Road, Saskatoon, SK S7N 2Z4, Canada; kja782@mail.usask.ca (K.J.H.A.); mem510@mail.usask.ca (M.E.M.); csf876@mail.usask.ca (C.F.); 2 Department of Orthopedics, University Medical Center Utrecht, Heidelberglaan 100, 3585 CX Utrecht, The Netherlands; j.f.f.hooningvanduijvenbode-2@umcutrecht.nl (J.F.F.H.v.D.); hcvogely@xs4all.nl (H.C.V.); h.h.weinans@umcutrecht.nl (H.W.); b.c.h.vanderwal@umcutrecht.nl (B.C.H.v.d.W.); 3 Department of Nuclear Medicine, University Medical Center Utrecht, Heidelberglaan 100, 3585 CX Utrecht, The Netherlands; b.vanderwildt@umcutrecht.nl (B.v.d.W.); a.j.poot@umcutrecht.nl (A.J.P.); m.lam@umcutrecht.nl (M.G.E.H.L.); 4 Department of Medical Microbiology, University Medical Center Utrecht, Heidelberglaan 100, 3585 CX Utrecht, The Netherlands; j.vanstrijp@umcutrecht.nl; 5 Department of Pathology, University Medical Center Utrecht, Heidelberglaan 100, 3585 CX Utrecht, The Netherlands; p.g.j.nikkels@umcutrecht.nl; 6 Department of Biomechanical Engineering, Delft University of Technology, Mekelweg 2, 2628 CD Delft, The Netherlands

**Keywords:** antibacterial strategies, biofilm, antibodies, radioimmunotherapy, targeted radiation

## Abstract

Background: the primary challenge with implant infections is the formation of biofilm, which harbors dormant bacteria that reduce the effectiveness of antibiotics and amplify antibiotic resistance, exacerbating the global antimicrobial resistance crisis. A potential novel treatment strategy is radioimmunotherapy, which uses antibodies linked to radioisotopes to deliver targeted radiation to the bacteria and biofilm. We describe the first in vivo use of targeted radiation therapy, employing Actinium-225 (α-radiation) and Lutetium-177 (β-radiation) labeled antibodies to treat a *Staphylococcus aureus* biofilm-associated intramedullary implant infection. Untargeted radiation in the form of unbound radionuclide treatment was also evaluated. Methods: to assess therapeutic efficacy, bacterial counts were performed on implant and surrounding bone after seven days of follow-up. Biodistribution was evaluated using SPECT/CT and ex vivo gamma counting. Results: radioimmunotherapy using an antibody against wall teichoic acid which was labeled with Actinium-225 and Lutetium-177 achieved bacterial reductions between 45% and 93% on the implant and surrounding bone. Surprisingly, a similar antimicrobial effect was observed with unbound Actinium-225 treatment reducing the bacterial load by 80% on the implant and 98% in the surrounding bone. Indications of maximum tolerated dose (MTD) with Lutetium-177 labeled antibodies were observed through hepatic and renal function evaluations. Conclusions: These results should be interpreted in the context of the study’s constraints, particularly the limited animal sample size. Nonetheless, the results suggest that in vivo applied radiation may help reduce a biofilm-associated infection at the implant site as well as in the surrounding bone. These findings encourage further investigation into the use of targeted and non-targeted radiation, potentially combined with antibiotics, to develop effective strategies for eradicating biofilm-associated implant infections.

## 1. Introduction

Biofilm-associated infections are a well-known and serious complication in present-day implant surgery, with approximately 80% of implant-related infections linked to bacterial biofilms [1]. Among these, the *Staphylococcus aureus* is the most prevalent pathogen, responsible for 30% of all biofilm-associated implant infections [2,3].

Biofilm is a fixed matrix containing exopolysaccharides, fibrins and lipoproteins in which microbial communities reside, and it has the ability to irreversibly attach to implants surfaces [4]. Biofilm formation is a complex process involving an attachment phase, microcolony formation phase, and maturation and dispersion phase [5]. Biofilm can exhibit tolerance to the host immune system by the inhibition of phagocytosis, opsonization avoidance and inactivation of the complement system. The formation of mature biofilm, with high bacterial density, is associated with the chronicity of implant infections [6]. Moreover, in chronic biofilm-associated implant infections, persister cells can regrow and cause infection relapse after antibiotic therapy is discontinued [7]. Older biofilms are more tolerant to antibiotics, with minimal inhibitory concentrations 10 to 1000 times higher than those for planktonic bacteria [8].

Moreover, in these biofilms, mutations are induced by spontaneous, antibiotic- or stress-induced mutagenesis. Owing to the increased number of bacteria that can survive within the biofilm, this layer serves as a reservoir for resistant bacterial clones, which paves the way for the development of antimicrobial resistance [9]. Given the challenges posed by biofilm and the growing threat of antimicrobial resistance, new bactericidal strategies must be developed.

Radioimmunotherapy (RIT) is the application of antibodies labeled with radionuclides to provide targeted radioactive therapy. This type of treatment is already a known and an applied treatment option in oncology and could be used to treat biofilm-associated implant infections, in case a specific antibody is used that targets the biofilm [10]. Teichoic acids are surface polymers found on the bacterial cell wall of most Gram-positive bacteria, such as the *S. aureus*. If bound to a peptidoglycan, it is called wall teichoic acid (WTA), a glycopolymer also expressed in the extracellular matrix of biofilm [11,12]. WTA has been demonstrated to be an excellent target for 4497-IgG1 antibody (anti-β-GlcNAc WTA) in vitro and in vivo and can labeled with alpha or beta radioisotopes to create targeted radioimmunotherapy for Gram-positive bacterial infections [13]. Alpha and Beta radiation have tissue penetration depth of 85 µm and 1–2 mm, respectively [14,15,16]. In combination with pathogen-specific antibodies, cytotoxic radiation can be delivered at the infection site, resulting in bacterial cell death through single- or double-strand DNA breaks. Additionally, oxidative stress can contribute to bacterial death through radiation-induced reactive oxygen species formation [17]. In vitro antimicrobial efficacy against the *S. aureus* with the 4497-IgG1 antibodies labeled with Bismuth-213 (alpha-radiation, half-life of 45.6 min), Actinium-225 (alpha-radiation, half-life 9.9 days) and Lutetium-177 (beta-radiation, half-life 6.7 days), has been documented [18,19]. Likewise, in vitro and in vivo antifungal efficacy against *Cryptococcus neoformans* fungi with 18B7-IgG1 labeled with Bismuth-213 and Rhenium-188 (beta-radiation, half-life of 16.9 h) has been reported [20,21].

In this preliminary study, we present a proof of principle for the application of radionuclides for treatment of a biofilm associated infection of an intramedullary implant. Therefore, we used Wistar Han rats with a surgically placed femur implant colonized with a maturated *S. aureus* biofilm, mimicking a chronic implant infection. Radionuclides Actinium-225 and Lutetium-177 were used with and without the antibody against *S. aureus* peptidoglycan β-GlcNAc WTA, which is also highly enclosed within the biofilm. We herewith tested if the application of different doses of radioimmunotherapy exerts a relevant antimicrobial effect on the implant-associated infection and used SPECT/CT and gamma counting to determine specific targeting and biodistribution of the radionuclides. Lastly, we conducted a short-term tolerance assessment of the radioimmunotherapy.

## 2. Results

### 2.1. In Vivo Experiment

**Figure A1:**
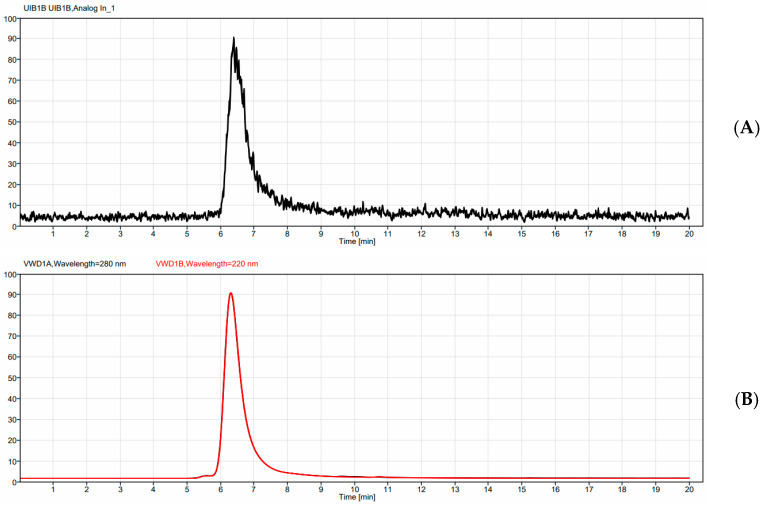
HPLC characterization shows radiopurity of Actinium-225 labeled 4497-antibody with DOTA conjugation. HPLC assessment was performed directly after radiolabeling on the day of administration (**A**) HPLC radio-trace of the ^225^Ac-4497 (**B**) HPLC UV-trace of ^225^Ac-4497.

**Figure A2:**
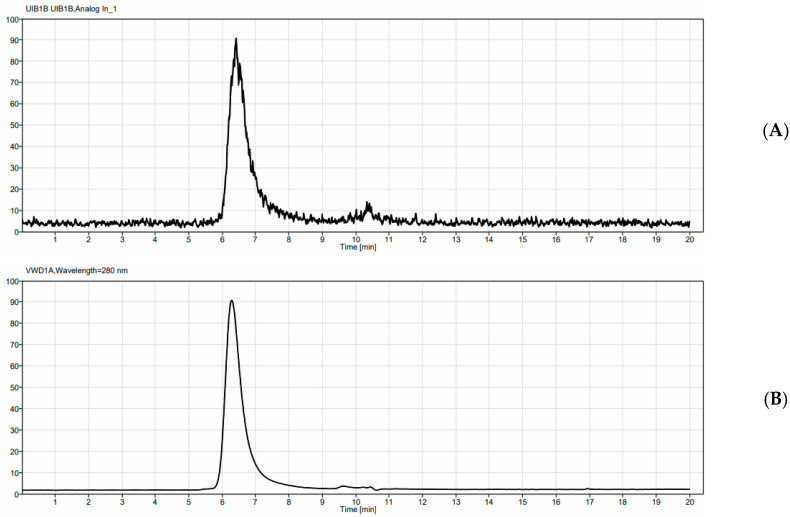
HPLC characterization shows radiopurity of Lutetium-177 labeled 4497-antibody with DOTA conjugation. HPLC assessment was performed directly after radiolabeling on the day of administration (**A**) HPLC radio-trace of the ^117^Lu-4497 (**B**) HPLC UV-trace of ^177^Lu-4497.

Bacterial counts on the autoclaved implants (n = 3) and biofilm growth medium showed no detectable CFU. The biofilm infected implants had a mean bacterial count of 1.1 × 10^8^ CFU (n = 5) prior to implantation. The radiopurity of the administered radiolabeled 4497-DOTA conjugate for the ^225^Ac-4497 treatment groups and ^177^Lu-4497 treatment groups was 88% and 98%, respectively, as determined by iTLC. HPLC characterization of ^225^Ac-4497 and ^177^Lu-4497 antibodies is shown in Figure A1 and Figure A2. RadioHPLC traces confirm that the antibody conjugate remained intact during the labeling process. Stability studies on ^225^Ac-4497 show that samples maintained in human serum show higher stability than those in PBS at 37 °C with the former remaining 84% stable over 7 days versus 67% stable for the PBS sample.

**Figure A3:**
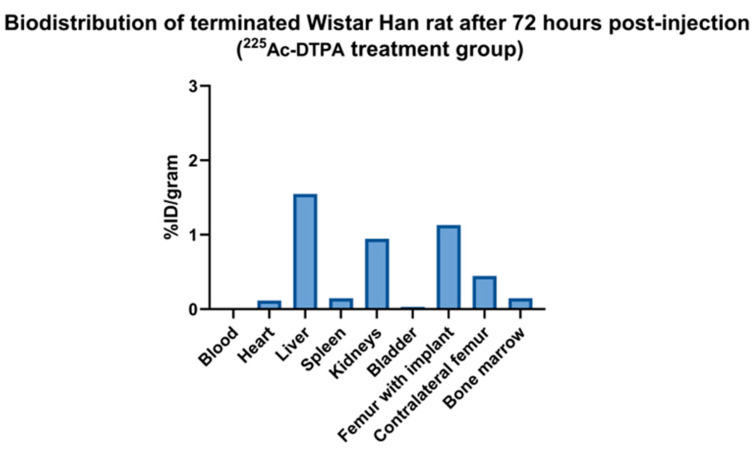
Ex vivo biodistribution of the prematurely terminated rat (^225^Ac-DTPA treatment group at 72 h after administration, shows already targeting with accumulation in the infected femur with implant. Due to behaviors consistent with pica, which escalated with continuous ingestion of the sutures this Wistar han rats was terminated before the 7-day follow-up. After termination the organs were retrieved and measured for accumulated radioactivity (%ID/gram) via gamma-counting. The %ID/gram for the liver, kidney and femur with implant was 1.55, 0.95 and 1,13, respectively.

All rats, except for one, completed the experiment. The affected rat in the Actinium-DTPA intervention group developed behaviors consistent with pica, which escalated into continuous ingestion of the sutures. These adverse effects may be associated with the administration of high-dose buprenorphine, potentially contributing to the observed symptoms. After consultation with the veterinarian, the rat was euthanized 72 h post-injection with ^225^Ac-DTPA. Subsequently, CFU count and biodistribution were performed. The CFU count on the implant and on the surrounding bone was 6.2 × 10^7^ and 5.8 × 10^7^, respectively. The biodistribution results are shown in Figure A3.

### 2.2. Treatment of a Biofilm-Associated Implant Infection with Radioimmunotherapy

**Figure 1:**
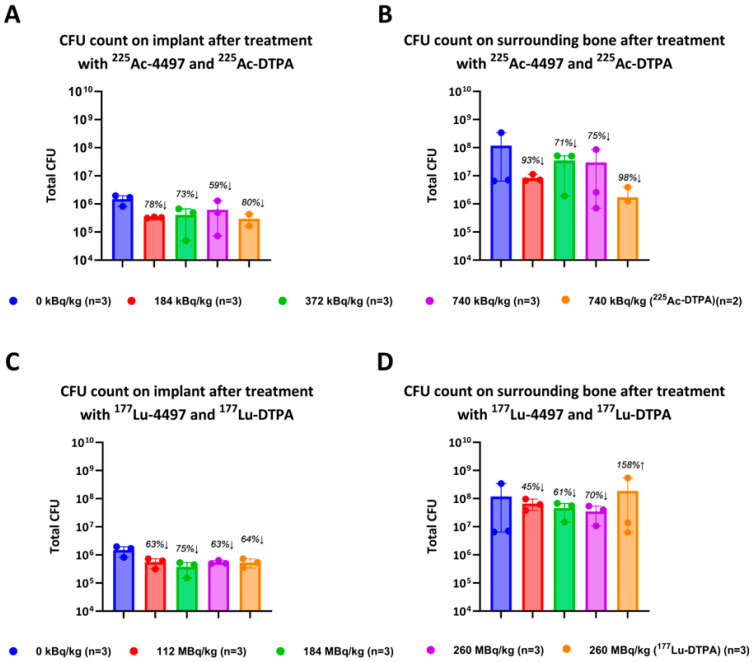
In vivo antimicrobial activity after seven days of follow-up. Wistar han rats operated on with an intramedullary femoral implant infected with three-days old maturated biofilm a treated with 3 different doses of (**A**,**B**) Actinium-225 labeled antibodies (^225^Ac-4497) and unbound radionuclide treatment with Actinium-225 (^225^Ac-DTPA). Or (**C**,**D**) Lutetium-177 labeled antibodies (^177^Lu-4497) and unbound radionuclide treatment with Lutetium-177 (^117^Lu-DTPA).

After seven days of follow-up post ^225^Ac-4497 administration, the percentage reduction in bacterial load on the implant for the 184 kBq/kg, 372 kBq/kg and 740 kBq/kg treatment groups, compared to the control group was 78%, 73% and 59%, respectively (Figure 1A). The ^225^Ac-DTPA (740 kBq/kg) treatment group meant as a non-targeted negative control showed unexpectedly an 80% bacterial load reduction on the implant compared to the control group (Figure 1A).

The antimicrobial effect of ^225^Ac-4497 on the total amount of bacteria in the surrounding bone was slightly greater than its effect on the implant, although the total amount of bacteria in the bone was approximately a factor 100 higher that on the implant. The percentage reduction in bacterial load in the surrounding bone for the 184 kBq/kg, 372 kBq/kg and 740 kBq/kg treatment groups was 93%, 71% and 75%, respectively (Figure 1B). Again, the ^225^Ac-DTPA (740 kBq/kg) treatment group showed the greatest bacterial load reduction on the surrounding bone with 98% compared to the control group.

After seven days of follow-up post ^177^Lu-4497 administration, the percentage reduction in bacterial load on the implant for the 112 MBq/kg, 184 MBq/kg and 260 MBq/kg treatment groups, compared to the control group was 63%, 75% and 63%, respectively (Figure 1C). The ^177^Lu-DTPA (260 MBq/kg) treatment group showed a 64% bacterial load reduction on the implant compared to the control group (Figure 1C).

The antimicrobial effect of ^177^Lu-4497 on the total amount of bacteria in the surrounding bone was lower than the effect observed at the implant. The percentage reduction in bacterial load in the surrounding bone for the 112 MBq/kg, 184 MBq/kg, and 260 MBq/kg treatment groups was 45%, 61%, and 70%, respectively (Figure 1D). The ^177^Lu-DTPA (260 MBq/kg) treatment group showed a 158% increase in bacterial load in the bone compared to the control group.

All individual CFU results can be found in Table A1 and Table A2.

### 2.3. Ex Vivo Biodistribution and SPECT/CT Imaging

**Figure 2:**
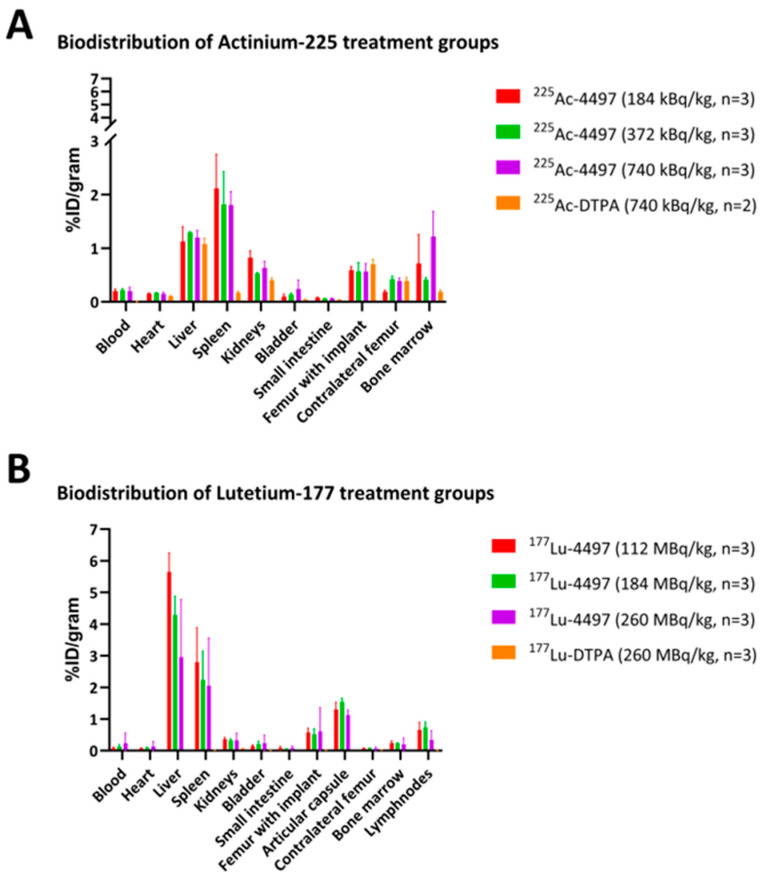
Ex vivo biodistribution after the 7 days follow-up. (**A**) ^225^Ac-4497 treatment and the ^225^Ac-DTPA treatment groups and (**B**) ^177^Lu-4497 and ^117^Lu-DTPA treatment groups. There is no significant difference in accumulation between the femur with implant of the ^225^Ac-4497 treatment groups (mean %ID/gram: 0.576, n = 9) and the ^225^Ac-DTPA treatment group (mean %ID/gram: 0.546, n = 2). *p* = 0.781.

After seven days gamma counts were determined for all organs. For all ^225^Ac-4497 treatment groups, the liver and spleen showed the greatest accumulation, given in percentage of the injected dose per gram of tissue (%ID/gram) (Figure 2A). The ^225^Ac-DTPA (740 kBq/kg) treatment group showed accumulation only in the liver and no accumulation in the spleen, in comparison to the ^225^Ac-4497 treatment groups. The mean %ID/gram ± SD of the femur with biofilm-infected implant after seven days of follow-up for the 184 kBq/kg, 372 kBq/kg, and 740 kBq/kg ^225^Ac-4497 treatment groups was 0.59 ± 0.01, 0.57 ± 0.16 and 0.56 ± 0.15, respectively. The mean %ID/gram of the femur with biofilm-infected implant for the ^225^Ac-DTPA treatment group (740 kBq/kg) was 0.70 ± 0.19 (Figure 2A).

Similarly, for all ^177^Lu-4497 treatment groups, the liver and spleen showed the highest %ID/gram, except for the ^177^Lu-DTPA (260 MBq/kg) treatment group (Figure 2B). The mean %ID/gram ± SD of the femur with biofilm-infected implant after seven days of follow-up for the 112 MBq/kg, 184 MBq/kg, and 260 MBq/kg ^177^Lu-4497 treatment groups was 0.57 ± 0.14, 0.52 ± 0.17 and 0.61 ± 0.75), respectively. The mean %ID/gram of the femur with biofilm-infected implant for the ^177^Lu-DTPA treatment group (260 MBq/kg) was 0.02 ± 0.001 (Figure 2B). The mean %ID/gram ± SD of the articular capsule for the ^177^Lu-DTPA treatment group (260 MBq/kg) was 0.01 ± 0.001 (Figure 2B).

Significant difference is found in accumulation between the femur with implant across all doses of the ^177^Lu-4497 treatment groups (mean %ID/gram: 0.568, n = 9) and the femur with implant of the ^117^Lu-DTPA treatment group (mean %ID/gram: 0.017, n = 3). *p* = 0.041. Also, significant difference in accumulation between the articular capsule (mean %ID/gram: 1.323, n = 9), and the femur with implant across all doses of the ^177^Lu-4497 treatment groups. *p* = 0.0001.

**Figure 3:**
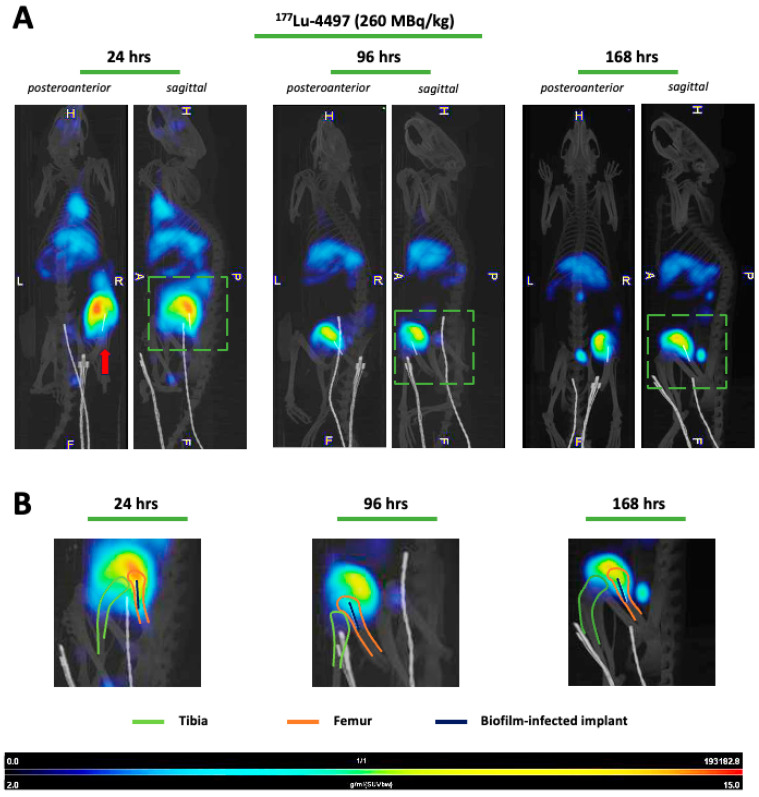
SPECT/CT analyses with ^177^Lu-4497 (260 MBq/kg) show specific accumulation after 24, 96 and 168 h. Posteroanterior and sagittal planes are depicted. H = head, L = left-side, R = right-side, P = posterior side, A = anterior side, and F = feet. The red arrow indicates the biofilm-infected implant site. The green square highlights the close-up shown in section B. (**A**) With the 4497-IgG1 antibody (anti-β-GlcNAc WTA) labeled with Lutetium-177, specific bacterial targeting and uptake are seen across all timepoints. (**B**) Close-up of the sagittal plane displaying the position of tibia, femur and biofilm-infected implant. Most accumulation is observed in the knee joint and articular capsule, distal to the implant infection.

**Figure 4:**
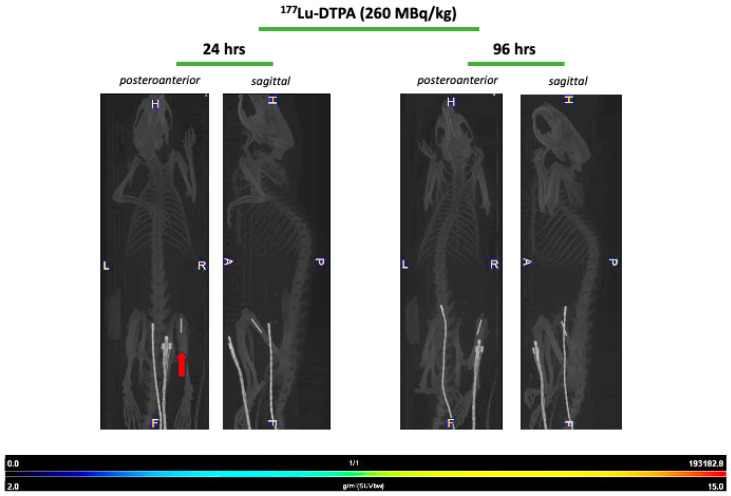
SPECT/CT analyses with ^177^Lu-DTPA (260 MBq/kg) show no accumulation after 24 and 96 h. Wistar han rats treated with ^177^Lu-DTPA (260 MBq/kg, n = 2) were scanned after 24- and 96 h post-injection for biodistribution assessment. Posteroanterior and sagittal planes are depicted. H = head, L = left-side, R = right-side, P = posterior side, A = anterior side, and F = feet. The red arrow indicates the biofilm-infected implant site. Unbound radionuclide treatment with Lutetium-177 showed no visual accumulation after 24 and 96 h. SPECT/CT analysis after 168 h was deemed unnecessary.

Imaging with SPECT/CT was performed only for the ^177^Lu-4497 treated rats. Accumulation of radiolabeled antibodies was observed in the knee joint and articular capsule distal to the implant infection (Figure 3). Therefore, the articular capsule was measured as well with the gamma counter for only the Lutetium-177 study. The mean %ID/gram of the articular capsule after seven days of follow-up for the 112 MBq/kg, 184 MBq/kg and 260 MBq/kg treatment groups was 1.30 ± 0.23, 1.54 ± 0.12 and 1.13 ± 0.16, respectively. The accumulation of radiolabeled antibodies in the knee joint and articular capsule distal to the implant infection for the ^177^Lu-DTPA treatment groups (without antibody, serving as a negative control) was negligible (Figure 4).

### 2.4. Short-Term Toxicity

**Figure 5:**
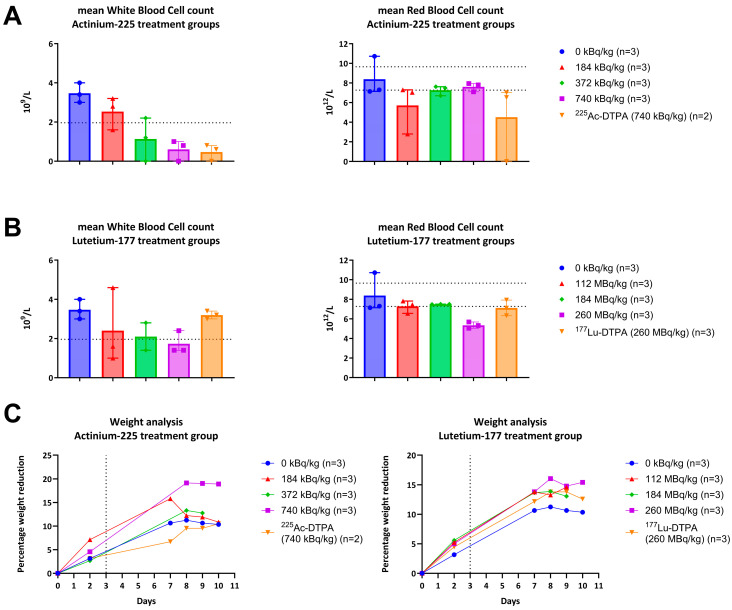
White blood cell count (WBC), red blood cell and weight analysis after 1 week of follow-up. (**A**) Dotted line resembles the lowest normal value for WBC: 1.96 × 10^9^/L. (**B**) Dotted line resembles the normal values for RBC: 7.27–9.65 × 10^12^/L. (**C**) Vertical dotted line resembles the timepoint of radioimmunotherapy administration. No animals in all treatment groups shows a mean percental weight reduction of more than 20%, which was the humane endpoint of this in vivo study.

Values of the short-term toxicity assessment from the control group were consistent with normal ranges and showed a WBC count and RBC count of 3.5 × 10^9^/L and 8.4 × 10^12^/L, respectively (Figure 5).

For the ^225^Ac-4497 treatment groups, an inverse dose-dependent relationship was found with the WBC count assessment, while a minimal effect was observed with the RBC count assessment. After seven days of follow-up, the WBC count (normal range: 1.96–8.25 × 10^9^/L) showed mean values for the 184 kBq/kg, 372 kBq/kg and 740 kBq/kg treatment groups of 2.6 × 10^9^/L, 1.2 × 10^9^/L and 0.6 × 10^9^/L, respectively. The ^225^Ac-DTPA treatment group showed a WBC count of 0.7 × 10^9^/L (Figure 5A). The RBC count (normal range: 7.27–9.65 × 10^12^/L) showed mean values for the 184 kBq/kg, 372 kBq/kg and 740 kBq/kg treatment groups of 5.8 × 10^12^/L, 7.2 × 10^12^/L and 7.6 × 10^12^/L, respectively. The ^225^Ac-DTPA treatment group showed an RBC count of 6.8 × 10^12^/L (Figure 5A).

For the ^177^Lu-4497 treatment groups, an inverse dose-dependent relation was found with the WBC count assessment. While a minimal effect was observed with the RBC count assessment. After seven days of follow-up, the WBC count showed mean values for the 112 MBq/kg, 184 MBq/kg and 260 MBq/kg treatment groups of 2.4 × 10^9^/L, 2.1 × 10^9^/L and 1.7 × 10^9^/L, respectively. The ^177^Lu-DTPA treatment group showed a WBC count of 3.2 × 10^9^/L (Figure 5B). The RBC count showed mean values for 112 MBq/kg, 184 MBq/kg and 260 MBq/kg treatment groups of 7.3 × 10^12^/L, 7.5 × 10^12^/L and 5.3 × 10^12^/L, respectively. The ^177^Lu-DTPA treatment group showed an RBC count of 7.2 × 10^12^/L.

For both the Actinium-225 and Lutetium-177 studies, all subjects experienced weight loss following surgery. After seven days of follow-up, no Wistar Han rat experienced a weight reduction of 20% from initial weight in any of the groups (Figure 5C).

**Figure 6:**
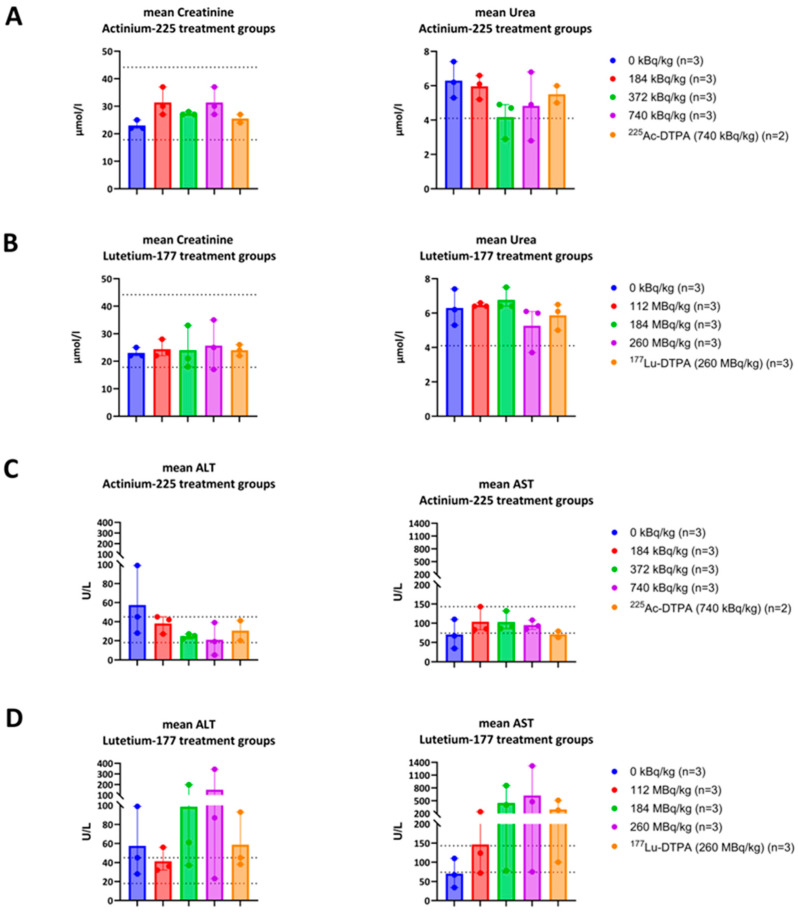
Short-term toxicity assessment after liver and kidney function analyses. (**A**,**B**) The dotted lines resemble the normal range of creatinine 17.7–44.1 μmol/L, and the highest normal value for urea: 4.1 mmol/L. (**C**,**D**) The dotted lines resemble the normal range of alanine transaminase (ALT): 18–45 U/L and for aspartate transferase (AST): 74–143 U/L.

The kidney panel analysis showed normal creatinine values but increased urea values for all treatment groups (Figure 6). The liver panel analysis showed no increase in ALT and AST values for both ^225^Ac-4497 or ^225^Ac-DTPA treatment groups. However, the ^177^Lu-4497 treatment groups showed a dose-dependent increase in ALT and AST. The ^177^Lu-DTPA treatment group showed no increase in ALT values and minimal increase in AST values (Figure 6).

### 2.5. Histology

**Figure 7:**
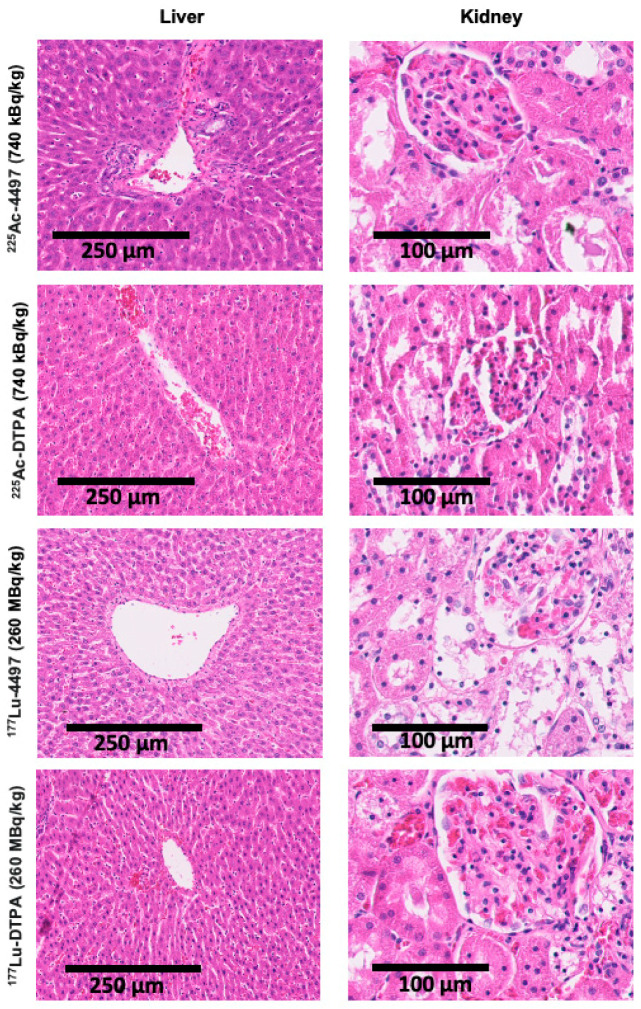
Histopathological analyses of the liver and kidney show minor damage after seven days of follow-up. Haematoxylin and eosin staining was used to assess organ damage with targeted and non-targeted Actinium-225 (alpha-radiation) and Lutetium-177 (beta-radiation) treatment after seven days of follow-up.

After a seven-day follow-up, histological assessment of the liver and kidney showed proximal tubule cell injury in the kidneys and incidental hepatocyte cell death around the central vein in the liver for both the ^225^Ac-4497 (740 kBq/kg) and ^225^Ac-DTPA (740 kBq/kg) treatment groups (Figure 7). For the ^177^Lu-4497 (260 Mbq/kg) treatment group, proximal tubule cell injury in the kidneys was characterized by the presence of proteinaceous debris in Bowman’s space. However, no histopathological changes were observed in the liver. No histopathological signs were observed in the kidney or liver for the ^177^Lu-DTPA (260 Mbq/kg) treatment group (Figure 7).

## 3. Discussion

There is substantial evidence that alpha-radiation and beta-radiation can eradicate bacteria and fungi in vitro and in vivo [18,21]. Including our previous results with the same 4497-antibody on *S. aureus* bacteria, displaying 99% and 99.99% killing when labeled with Actinium-225 and Lutetium-177 radionuclide, respectively [19].

However, no in vivo radioimmunotherapy treatments for surgical subjects with biofilm-associated implant infections have been attempted. This study is the first to apply radioimmunotherapy (RIT) and unbound radionuclide treatment in Wistar Han rats with an intramedullary femur implant infected with three-day matured *S. aureus* biofilm. This study was designed as an exploratory in vivo proof-of-concept investigation to evaluate the feasibility and biodistribution of pathogen-specific radioimmunotherapy.

Due to the dormant character of the biofilm, sufficient infection development was ensured by allowing a three-day incubation period before administering the RIT and the unbound radionuclide treatment. Our results indicate that targeted alpha-radiation (^225^Ac-4497) has a combined small antimicrobial effect on the implant and surrounding bone, whereas beta-radiation (^177^Lu-4497) had a similar effect on the implant but almost no antimicrobial efficacy on the surrounding bone after the seven-day follow-up period. A significant observation in this study was the shift in RIT accumulation to the joint and articular capsule of the infected leg as a consequence of new infection onset in the joint (Figure 3). For effective radioimmunotherapy, precise targeting is essential, as is defining an antimicrobial dose that is both therapeutically effective and minimally toxic. Given that alpha radiation has a tissue penetration depth of only 47–85 µm, whereas beta radiation penetrates approximately 1–2 mm, achieving accurate radionuclide localization is critical [15,16,17]. Any redistribution of the radiopharmaceutical could therefore alter the treatment effect. The observed redistribution might have resulted in reduced radiation exposure to the deeper-positioned infected implant and bone. As a consequence, we did not report the full potential of the antimicrobial effect of RIT on a biofilm-associated implant infection. The antimicrobial effects on the implant and surrounding bone were minimal (ranging from a 45–93% reduction) and did not achieve the desired bactericidal effects, which typically requires a 99.9% reduction (a 3-log reduction) [22]. Initially, it was hypothesized that unbound radionuclide treatment would serve as a negative control, leading to rapid clearance of the radioisotope, and consequently, highlighting the targeting efficiency of the 4497-antibody. This was observed during treatment with unbound Lutetium-177, which showed no uptake throughout the study duration (Figure 4). No SPECT/CT analyses of ^225^Ac-4497 were performed due to the low gamma emission of Actinium-225, which makes sufficient imaging challenging. However, since the same 4497-antibody was used, a similar biodistribution was expected. Surprisingly, the unbound radionuclide treatment group using Actinium-225 also performed in the same range as RIT, achieving an 80% CFU reduction on the implant. Even more unexpectedly, this treatment group demonstrated a 98% CFU reduction in the surrounding bone, approaching nearly a 1-log reduction (99%). This finding was supported by the comparable ex vivo biodistribution accumulation of the ^225^Ac-DTPA and ^225^Ac-4497 in the femoral bone surrounding the implant (Figure 2A) and of the prematurely terminated Wistar Han rat (Figure A3). Additionally, this femoral accumulation was further corroborated by the observed leukopenia of the ^225^Ac-DTPA treatment group (Figure 5A). It is important to note that, in this preliminary investigation, ^225^Ac-DTPA was utilized primarily as a reference control to define baseline biodistribution patterns and quantify non-specific tissue accumulation, rather than as a comparator for therapeutic efficacy.

Actinides, such as the radionuclide Actinium-225, have been shown to have a high affinity for bone glycoproteins [23,24]. Similar to lanthanides, Actinium ions can substitute for calcium in the hydroxyapatite matrix because of their similar ionic radii and chemical properties [25,26]. This can occur particularly in cases of increased bone turnover, such as after bone implant surgery or during a local bone infection.

Presumably, three potential mechanisms may explain this unexpected accumulation. First, the instability of the ^225^Ac-DTPA complex could result in the release of free ^225^Ac^3+^, which may then accumulate in the infected femur (Figure 2A). This phenomenon was already observed in a mouse model [27]. Second, the intact ^225^Ac-DTPA complex itself may have organ-specific affinity. Davis et al. (1999) reported that Actinium-225 bound to a CHX-DTPA complex preferentially accumulates in the liver and bone compared to other organs five days post-injection [28]. Consistent with this, increased liver uptake of ^225^Ac-DTPA was also observed in the present study (Figure 2A). Third, a combination of both free isotope release and organ-specific uptake of the intact complex may contribute to the observed distribution.

As a result of its bone-targeting properties, the ^225^Ac-DTPA treatment localized to the entire femoral bone near the biofilm-infected implant, leading to the greatest observed reduction in CFU. Interestingly, despite the use of a limited small-animal sample size, the results suggest in vivo evidence of modest antimicrobial efficacy through radiation. Further research is needed to elucidate the in vivo stability of conjugating molecules with Actinium-225 and the mechanisms underlying Actinium-225 deposition in bone for its potential future application in biofilm-associated implant infections in larger animal studies.

The biodistribution of both ^225^Ac-4497 and ^177^Lu-4497 treatment groups shows greater uptake in the liver and spleen, which is expected due to the fenestrated capillaries with large paracellular pores and sinusoids to facilitate convective transport of the radionuclide-antibody conjugate [29]. As observed from the SPECT/CT analyses (Figure 3A), there is substantial accumulation of ^177^Lu-4497 (260 MBq/kg) at the right infected leg. However, it seems that the highest uptake occurs in the knee joint and articular capsule, as was confirmed with biodistribution analysis and SPECT/CT analyses (Figure 2B and Figure 3B). It can be confidently stated that during implantation, the joint and the surgical tract became infected with the same *S. aureus* AH4802 bacteria, resulting in the accumulation of the radionuclide-antibody conjugate in these locations. Given the fact that this distally located tissue is likely to be highly vascularized, the bacteria in the arthritic tissue likely take away a large portion of the ^177^Lu-4497 radionuclide-antibody conjugate. Unlike the application of RIT in tumor models, postoperative surgical effects and bacterial dispersion leading to new infection onset are factors that influence the biodistribution and may have affected the treatment efficacy of RIT in this model. The development of infection within the close-by joint and surgical tract creates a competitive environment in which radiolabeled antibodies may preferentially bind to the most accessible site of infection.

Regarding RIT with Actinium-225 and Lutetium-177, the bacterial reduction across all treatment groups was approximately within the same range for the implant and the surrounding bone. However, the results demonstrate that ^225^Ac-4497 (740 kBq/kg) treatment group performed best of all RIT treatments with 78% and 93% CFU reduction on the implant and surrounding bone despite the accumulation shift and even under the challenge of low WBC count (Figure 5A). Combined with the unbound Actinium-225 treatment group, this possibly suggests that alpha radiation exerts a greater initial antimicrobial effect in vivo compared to beta-radiation at the doses used.

Single- and double DNA strand breakage, as well as the formation of ROS within the bacteria, are both key mechanisms underlying the application of radioimmunotherapy for infections [18,19]. Besides these intended effects, ionizing particles can also have a negative impact on physiological systems, including parts of the immune system. The decay of Actinium-225 releases four alpha-particles with energies ranging from 5.8 to 8.4 MeV [17]. Due to this high linear energy transfer, a dose-dependent negative trend was observed in the white blood cell counts, compromising the host’s first line of defense within the innate immune system against the induced biofilm-associated implant infection. As the linear energy transfer of Lutetium-177 (0.497 MeV) is approximately tenfold lower than for Actinium-225, the treatment and systemic effects are expected to be milder [14,15]. Despite these mild antibacterial effects, a dose-dependent decrease in the white blood cell count was observed, similar to the ^225^Ac-4497 treatment groups (Figure 5A). There is evidence suggesting that these effects are transient and typically return to baseline over time [30,31]. Based on initial data, the ^177^Lu-4497 treatment group exhibited a dose-dependent increase in ALT and AST, unlike the ^225^Ac-4497 treatment group. This suggests that short-term tolerability for beta-radiation is achieved at 112 MBq/kg for Wistar Han rats. Because increases in aminotransferases can occur before visible tissue changes, no significant histopathological alterations were observed in the present study. In contrast, alpha-radiation treatment did not lead to increased liver or kidney function markers, suggesting that the maximum administered dose in the present study was still well tolerated after one week. No weight loss exceeding 20% was observed in this study. While some of the weight loss may be related to radiation exposure, it appears to be limited, especially considering that the rats also underwent major surgery with a biofilm-infected implant, which can all induce stress responses and associated appetite suppression.

Animal experiments involving biofilms are complex. In an in vivo experiment with biofilms, one must account for the dynamic flow of antimicrobials, hypoxic conditions, and time-dependent effects. Additionally, the in vivo environment can trigger adaptive survival mechanisms in the biofilm, which may alter its metabolic rate, replication rate, or surface protein expression, potentially modifying antimicrobial effects [32].

In this study, a clinical setting was simulated with an intramedullary femur implant in which a chronic infection was mimicked by introducing a three-day maturated biofilm-associated infection. The small sample size (n = 3 per group) limits our ability to draw firm conclusions for each individual group. However, the combined data shows a modest antimicrobial effect with Actinium-225. Whether Actininium-225 should be used with or without an antibody targeting the bacteria and their biofilm may depend on the intended application. Hypothetically, radioisotopes known to accumulate in bone (without the conjugation to an antibody) could be applied for osteomyelitis treatment and should be further explored. Despite the limitations regarding radiochemical stability and small animal sample size, this study provides the first in vivo indication that RIT can localize and affect biofilm-associated implant infections, warranting further preclinical optimization.

Our infection model, in which an implant infection is induced on the same day of surgery, accompanied postoperative healing processes, limits our ability to truly replicate a chronic infection. Nonetheless, by inducing an implant infection solely with a mature biofilm, the challenges of biofilm-associated implant infections, such as inhibition of phagocytosis, opsonization avoidance, and inactivation of the complement system, are being mimicked, unlike with planktonic bacterial infections [7]. It would be valuable to pursue future research with alpha-emitting radionuclides with shorter half-life, such as Bismuth-212/213 or Lead-212, which would increase radiation emission per unit of time. This approach could potentially better account for the in vivo bacterial replication rate, which has been reported to be in the range of 1–3 h in humans [33]. In oncology, radioimmunotherapy (RIT) is often administered fractionally to enhance therapeutic success [34]. In this study, however, we used a single injection approach. Future RIT applications in surgical implant infection models could explore multi-injection regimens. Moreover, applying RIT in this surgical infection model introduces complexities in biodistribution, as the onset of bacterial arthritis may have competed with the implant infection site for antibody binding. Interestingly, co-injecting unlabeled antibodies could potentially increase the systemic circulation of radiolabeled antibodies by reducing splenic antibody uptake. This strategy may enhance radiolabeled antibody accumulation at the target site, thereby improving treatment efficacy [35,36,37]. Additionally, intra-articular injection of the radiolabeled antibodies could result in higher local concentrations at the implant location, which might improve the treatment effects as well. After identifying an in vivo stable radionuclide–antibody complex and determining the optimal administration method, long-term toxicity assessments of liver and kidney function and larger animal studies should be conducted to evaluate its clinical potential.

Importantly, at this early stage, as we seek to establish evidence for the antimicrobial potential of targeted radiation therapy, combining antibiotics with radioimmunotherapy may prove highly valuable for treating biofilm-associated implant infections. Ionizing radiation is not hindered by the physical barrier of the biofilm or the low metabolic rate of biofilm-embedded (dormant) bacteria. Radiation could disrupt the biofilm, potentially increasing bacterial sensitivity to antibiotics and leading to a synergistic effect. If such a combination therapy could lower the minimal inhibitory concentration (MIC) of the antibiotic required for biofilm eradication, we would be one step closer to minimizing the burden of biofilm-associated implant infections and antimicrobial resistance.

## 4. Materials and Methods

### 4.1. Study Design

A biofilm-associated infection with a femoral implant was induced in 27 Wistar Han Rats. The antimicrobial effects of three different doses of Actinium-225-labeled antibodies (^225^Ac-4497) and Lutetium-177-labeled antibodies (^177^Lu-4497), as well as unlabeled ^225^Ac and ^177^Lu (conjugated to DTPA), were evaluated after seven days of follow-up. A saline injection (0 kBq/kg) served as the control intervention. Primary study outcomes included antimicrobial efficacy on the implant and surrounding bone, determined by bacterial count CFU. Secondary study outcomes involved short-term systemic effects (blood analysis) and tissue response (histology). Additionally, specific targeting of the biofilm and biodistribution was assessed during follow-up using SPECT/CT analyses (^177^Lu-4497 only) and ex vivo via gamma-count analyses (both isotopes). The in vivo experiments were approved by the Animal Research Ethics Board of the University of Saskatchewan, Canada (protocol AUP20230036). All experiments were performed in accordance with institutional guidelines and regulations, and with the ARRIVE guidelines for reporting animal research [38].

### 4.2. Antibody Conjugation

Antibodies were made according to protocol as described in detail by de Vor et al. [13]. In short, the Mut (H+Y) HuIgG1-antiWTA-4497 antibody (4497-antibody) was produced by cloning the human constant regions into pcDNA3.4 vectors harboring the variable heavy (VH) and light (VL) chain sequences. These sequences, which were codon-optimized and contained KOZAK and HAVT20 signal peptides, were originally derived from B cells of patients infected with *S. aureus*. After transfection into EXPI293F cells, IgG1 antibodies were recovered using HiTrap protein A columns from the supernatant within 4–5 days. Following dialysis in PBS and filter sterilization, the antibodies were examined for aggregation and stored at a concentration of 7.68 mg/mL at 4 °C.

Conjugation of the 4497-IgG1 antibody to the bifunctional chelator S-2-(4-Isothiocyanatobenzyl)-1,4,7,10-tetraazacyclododecane tetraacetic acid (p-SCN-Bn-DOTA, Macrocyclics^TM^, Plano, TX, USA) was performed by exchanging the antibody’s storage buffer with NaHCO_3_ and Na_2_CO_3_ buffer solution that had been passed through a chelex-100 cation exchange resin to remove any advantageous metals (chelexed). A 0.5 mL 30 kDa molecular weight cutoff Amicon microconcentrator (Millipore, Burlington, MA, USA) was used to ensure complete exchange of the storage buffer; this process was repeated 10 times at 4 °C. A 10-fold molar excess of p-SCN-Bn-DOTA was prepared in NaHCO_3_ and Na_2_CO_3_ buffer solution immediately prior to use and then added to the antibody solution. This reaction mixture was incubated at 37 °C for 1.5 h under agitation. The 4497-DOTA mixture was then exchanged into a 0.15 M ammonium acetate buffer (chelexed) using a 30 kDa molecular weight cutoff Amicon microconcentrator, followed by centrifugation (10× *g* at 4 °C) [39].

### 4.3. Radiolabeling with Actinium-225 and Lutetium-177

0.037 MBq of ^225^Ac and 0.37 MBq of ^177^Lu per µg of 4497-DOTA conjugate were radiolabeled at 37 °C for 1 h (the pH was maintained at 7 throughout the radiolabeling process). Generally, for ^225^Ac (Oakridge National Labs, Oak Ridge, TN, USA) labeling, the desired amount of ^225^AcCl_3_ (37 mBq), dissolved in 0.01 M HCl, was added to a minimum volume of 0.15 M ammonium acetate, giving a 50 µL final volume. To this solution, 100 µg of antibody (47.6 µL) was added, and the solution was heated at 37 °C for 1 h. After, 3 µL of 5 mM DTPA solution was added to bind any free ^225^AcCl_3_. For ^177^Lu (McMaster University, Hamilton, ON, Canada), labeling was performed in a similar manner using 555 mBq of ^177^LuCl_3_ in 50 µL of 0.15 M ammonium acetate solution. 1.5 mg of 4497-DOTA conjugate (714 µL) was added to the radiometal and heated at 37 °C for 1 h. After, 3 µL of 5 mM EDTA solution was added to bind any free ^177^LuCl_3_. Silica gel strips (10 cm length, Agilent Technologies Inc., Santa Clara, CA, USA) were used to measure the radiopurity of the radiolabeled 4497-DOTA conjugate by instant thin layer chromatography (iTLC) with 0.5 mM EDTA as mobile phase, approximately 30 to 60 min before injection. The strips were cut in half, and each part was measured using a gamma counter (2470 Wizard2 Gamma counter, PerkinElmer, Waltham, MA, USA). The gamma counter was calibrated for either the Actinium-225 or Lutetium-177 emission spectrum, and only emissions within this range were counted in counts per minute (CPM). The CPM of the radiolabeled antibody was divided by the total sum of CPM from both strips and multiplied by 100 to calculate radiopurity.

Samples of each radio-conjugate were then run on an Agilent HPLC (Agilent Technologies Inc., Santa Clara, CA, USA) equipped with a UV detector monitoring at UV = 280, a BioScan radioactivity detector (Eckert & Ziegler, Valencia, CA, USA), and a TSKgel SuperSw2000, 4.6 mm I.D. X 30 cm, 4 µm SEC column (TOSOH Bioscience, Tokyo, Japan). An isocratic method was run for 20 min with a 150 mM Sodium Phosphate buffer, pH 7, as the eluant.

A stability study was carried out by taking purified ^225^Ac-4497 and diluting it in 90% human serum or 90% PBS and storing it at 37 °C. Aliquots were removed at 24, 48, 72, and 168 h and added to a 5 mM DTPA solution. Samples were then allowed to sit for 5 min before being run on iTLC strips as previously described.

**Figure 8:**
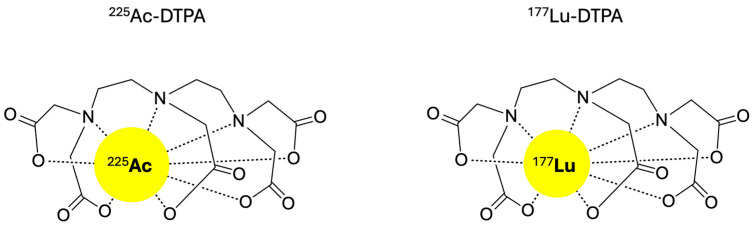
The chemical structures of ^177^Lu-DTPA and ^225^Ac-DTPA.

Evaluation of non-targeted radiation effects was performed with the unbound radionuclide treatment groups. Diethylenetriaminepentaacetic acid (DTPA) was used as a chelator for the unbound Actinium-225 and Lutetium-177 radionuclides [40]. Due to the multiple binding sites of this chelating molecule, it facilitates the formation of stable negatively charged in vivo complexes with trivalent metal ions such as Actinium-225 (Ac^3+^) and Lutetium-177 (Lu^3+^), thereby preventing the hydrolysis of free radiometals and facilitating their excretion through the kidneys (Figure 8). The hydrophilic DTPA was chosen over the more hydrophobic DOTA chelating molecule to reduce unwanted accumulation of the unbound Actinium-225 and Lutetium-177 ions. For the unbound radionuclide treatment groups, the administered radioactive dose was matched with the highest dose of the ^225^Ac-4497 (740 kBq/kg) and ^177^Lu-4497 (260 MBq/kg) radioimmunotherapy treatment groups.

### 4.4. Biofilm Cultures on Implants

In these experiments, the *S. aureus* USA300 LAC (AH4802) strain was used [41]. The bacteria were cultured at 37 °C overnight on tryptic soy broth (TSB). The overnight culture was then re-inoculated into fresh TSB medium and incubated for 3 h to allow logarithmic growth before use.

The implants were made of a titanium alloy (medical grade 23, ELI, Ti6AI4V) with a length of 10 mm and a width of 0.8 mm. The implants were produced at the Additive Manufacturing Lab (TU Delft, Delft, The Netherlands) using a selective laser melting machine (SLM-125, Realizer, Frankfurt, Germany). Grooves were incorporated into the implants to improve the attachment of biofilm upon insertion and the implants were autoclaved prior to culturing biofilm on the surface of these titanium implants. To further facilitate bacterial binding, 250 μL of human fibronectin (Sigma, Saint Louis, MO, USA) at a concentration of 20 μg/mL in carbonate–bicarbonate buffer was incubated with the implants overnight at 4 °C. An overnight culture with *S. aureus* bacteria was diluted to an OD600 of 1 and then diluted 1:10 in fresh biofilm growth medium (TSB containing 0.5% *w*/*v* glucose and 3% *w*/*v* NaCl). Subsequently, 500 μL of this suspension was transferred to the implant and incubated statically for 72 h at 37 °C. Every 24 h, 250 μL of the medium was replaced with fresh biofilm growth medium. Prior to implantation, the implants with three-day-old biofilms were washed three times to remove any planktonic bacteria.

### 4.5. Biofilm-Associated Implant Infection and Radioimmunotherapy

Twenty-seven male Wistar Han rats (Charles River laboratories, Montreal, QC, Cananda) of approximately 13 weeks old were operated to insert one femoral rod implant into the intramedullary canal of the right femur. Three days after the surgical procedure, radioimmunotherapy was administered intravenously via the tail.

In the Actinium-225 study, twelve Wistar Han rats were treated with the 4497-antibody radiolabeled with Actinium-225 (^225^Ac-4497). The doses per treatment group were 184 kBq/kg (n = 3), 372 kBq/kg (n = 3) and 740 kBq/kg (n = 3), respectively. Three rats received DTPA-bound Actinium-225 (^225^Ac-DTPA treatment group) as an unbound radionuclide treatment with an activity of 740 kBq/kg (n = 3).

In the Lutetium-177 study, twelve Wistar Han rats were treated with the 4497-antibody radiolabeled with Lutetium-177 (^177^Lu-4497). The doses per treatment group were 112 MBq/kg (n = 3), 184 MBq/kg (n = 3) and 260 MBq/kg (n = 3), respectively. Three rats received DTPA-bound Lutetium-177 (^177^Lu-DTPA treatment group) as an unbound radionuclide treatment with an activity of 260 MBq/kg (n = 3). Additionally, three rats were treated with saline, serving as a control group (0 kBq/kg).

The doses used for the Actinium-225 and Lutetium-177 treatment groups were guided by general insights from prior research within our group and studies involving radiolabeled antibodies. All rats were followed for seven days after radioimmunotherapy administration prior to termination. Food and water were provided ad libitum. Only in the Lutetium-177 study, SPECT/CT analyses were performed at 24 h, 96 h and 168 h post-injection for the ^177^Lu-4497 (260 MBq/kg) treatment group and ^177^Lu-DTPA treatment group (260 MBq/kg).

### 4.6. Implant Surgery

In short, all Wistar Han rats were anesthetized with isoflurane (induction dose: 5% and maintenance dose: 2–3%). Prior to the surgery, 2 mL NaCl 0.9%, slow-release buprenorphine (0.05 mg/kg) and meloxicam (2 mg/kg) were administered subcutaneously. After a straight midline incision on the right knee, the patella was repositioned to the medial side exposing the femoral condyles. The entrance to the medullary canal was established using a drill with a length of 1 cm and a diameter of 1 mm, creating an insertion hole between the two femoral condyles. The biofilm-infected implant was inserted, and the opening was then closed with bone wax (SMI). Absorbable sutures were used to reposition the patella back to anatomical position and to close the inner tissues. Non-absorbable sutures were used to close the skin. Slow-release buprenorphine (0.05 mg/kg) was administered every other day, and meloxicam (2 mg/kg) was given daily for three days post-surgery for pain management.

### 4.7. CFU Count Assessment

Prior to biofilm maturation, three randomly chosen autoclaved implants and the biofilm growth medium were evaluated for bacterial growth to ensure no introduction of other pathogens than the *S. aureus* USA300 LAC (AH4802) pathogen. Additionally, prior to implantation of the biofilm-infected implants, five randomly chosen implants were assessed for CFU after three days of biofilm maturation to evaluate initial bacterial load upon insertion. After seven days of follow-up, the biofilm-infected femur with implant was collected from all animals, and subsequently stored in ice until assessment. Before the CFU assessment, the femurs were rinsed with sterile PBS. Aseptically, the implant was separated from the bone and stored in 1 mL of sterile PBS. The surrounding femoral bone was cut into pieces and placed in 20 mL sterile PBS. A Kinematica Polytron^®^ (PT 10), Kinematica AG, Luzern, Switzerland) was used to homogenize the bone. Both implant and homogenized bone were sonicated for 10 min. Ten-fold serial dilutions were cultured on Colombia Blood Agar plates in duplicate. CFU was counted after overnight incubation in a 37 °C stove. The total number of bacteria in the bone and on the implant was calculated by multiplying the CFU/mL by the total volume of the sample.

### 4.8. Ex Vivo Biodistribution Assessment

Assessment of the biodistribution after seven days of follow-up was performed by removal of the organs and the implants with the surrounding femur immediately after euthanizing the rats with an overdose of isoflurane. Only in the Lutetium-177 study (^177^Lu-4497 and ^177^Lu-DTPA treatment groups), the articular capsule was also collected for ex vivo biodistribution assessment, as accumulation was observed due to new infection onset in the joint, with SPECT analyses. All samples were weighed. Radioactivity was measured with a gamma counter. Prior to the injection of interventions, a standard was created with one-tenth of the injected dose of each treatment group and was subsequently kept in 0.15 M ammonium acetate. These standards were counted in a gamma-counter at the same time as the collected organs and implants with the surrounding femur, allowing for decay correction. The standards were diluted by 10-fold for the ^225^Ac samples and 100-fold for the ^177^Lu samples to keep the standards in the linear range for the detector. The percentage of injected dose per gram (%ID/g) organs and implant was calculated by the following formula with Z representing the dilution factor (Z = 10 for ^225^Ac and Z = 100 for ^177^Lu):


$$\%\;\mathrm{Injected}\;\mathrm{dose}\;\mathrm{per}\;\mathrm{gram}\;(\%\;\frac{\mathrm{ID}}{\mathrm{gram}})\;=\;\frac{\mathrm{Counts}\mathrm{per}\mathrm{minute}\;(\mathrm{CPM})}{\mathrm{Organ}\mathrm{weight}\;(\mathrm{gram})\;\times \;(Z\;\times \;\mathrm{CPM}\;\mathrm{standard})}\;\times \;100$$

### 4.9. Short-Term Toxicity Assessment

Blood collection was performed by heart puncture and stored in tubes with Lithium Heparin (BD microtainer). Blood plasma was obtained by centrifuging the samples for 4 min at 10 G. Subsequently, all blood plasma samples were stored at −80 °C until no detectable radioactivity was measured prior to the liver panel analysis (creatinine and urea) and kidney panel analysis (AST and ALT). White blood cell count (WBC) and red blood cell count (RBC) were assessed directly after termination using a Beckman-Coulter Ac.T differential hematology analyzer (Beckman Coulter Inc., Brea, CA, USA).

### 4.10. Histological Processing

Histopathological analysis of radiation damage to the liver and kidney were investigated by fixing the organs in 4% *w*/*v* formaldehyde. Subsequently, the samples were dehydrated in an ethanol series. Thereafter, the samples were embedded in paraffin and cut into 6 µm thick slices using a sawing microtome (Leica, Nussloch, Germany). The slices were stained with hematoxylin and eosin (H&E). assessed the slices for organ damage. The kidney samples were evaluated for renal architecture, glomerular, and tubulointerstitial changes. The liver samples were evaluated for liver architecture, fibrosis, and hepatocellular changes.

### 4.11. Statistical Analysis

The aim of this study was to evaluate an in vivo antimicrobial effect of targeted radiation in a dose-dependent setting and to perform a tolerance assessment for Wistar Han rats. An arbitrary sample size of three was chosen per treatment group. Due to the small sample size, no statistical tests were performed on the antimicrobial efficacy. The percentage change in bacterial count (CFU) compared to the control treatment group was calculated and reported. The mean and range were reported in graphs using GraphPad Prism 7.4. An unpaired *t*-test was performed to assess differences in the ex vivo biodistribution assessment. All reported hematological parameters, kidney panel results (creatinine and urea), and liver panel results (AST and ALT) were compared to the clinical lab parameters provided by Charles River Laboratories [42].

## 5. Conclusions

The development of new antibacterial strategies is essential to tackle the burden of biofilm-associated implant infections and the emerging problem of antimicrobial resistance. In this preliminary study, radioimmunotherapy and unbound radionuclide treatment using Actinium-225 and Lutetium-177 was explored in a biofilm-associated implant infection model in Wistar Han rats. The data demonstrated effective in vivo bacterial targeting with RIT. However, the onset of a secondary infection in the joint and articular capsule led to a shift in RIT accumulation, which might have resulted in a weakened antimicrobial impact on the implant and bone. Notably, an even stronger antimicrobial effect was observed with unbound radionuclide treatment with Actinium-225 in comparison to antibody-bound RIT treatment groups after a seven-day follow-up. This proof-of-concept study on the in vivo application of targeted ionizing radiation therapy for biofilm-associated implant infections provides insights to guide improvements in future experiments and suggests that RIT, alone or combined with antibiotics, may contribute to the development of novel antimicrobial strategies and warrants evaluation in larger in vivo studies.

## Data Availability

The complete data supporting the findings of this study are available from the corresponding author upon reasonable request. This includes raw data, analysis scripts, and any other data.

## Abbreviations

The following abbreviations are used in this manuscript:

^177^Lu	Lutetium-177
^225^Ac	Actinium-225
4497-antibody	Mut (H+Y) HuIgG1-anti-Wall Techoic Acid-4497 antibody
ALT	Alanine Aminotransferase
AST	Aspartate Aminotransferase
CFU	Colony forming units
CPM	Counts per minute
DOTA	1,4,7,10-tetraazacyclododecane-1,4,7,10-tetraacetic acid.
DTPA	Diethylenetriaminepentaacetic acid
H&E	Hematoxylin and Eosin
HE-UHR-RM	High-energy Ultra High-Resolution Rat Mouse
IgG1	Immunoglobulin G subclass 1
MIC	minimal inhibitory concentration
OD_600_	Optical Density at 600 nanometers.
RBC	Red blood cell
RIT	Radioimmunotherapy
SPECT/CT	Single Photon Emission Computed Tomography/Computed Tomography
TSB	Tryptic Soy Broth
WTA	Wall Teichoic Acid
WBC	White Blood Cell
%ID	Percentage Injected Dose

## Appendix A

### Appendix A.2

**Table A1:** Actinium-225 treatment groups (^225^Ac-4497 and ^225^Ac-DTPA). The Individual mean Colony Forming units (CFU) after seven days follow-up.

	Implant (CFU)			Bone (CFU)		
	Rat #1	Rat #2	Rat #3	Rat #1	Rat #2	Rat #3
0 kBq/kg (n = 3)	1.7 × 10^6^	8.2 × 10^5^	1.95 × 10^6^	7.0 × 10^6^	3.4 × 10^8^	6.5 × 10^6^
184 kBq/kg (n = 3	3.15 × 10^5^	3.45 × 10^5^	3.3 × 10^5^	1.13 × 10^7^	7.1 × 10^6^	6.7 × 10^6^
372 kBq/kg (n = 3)	5.0 × 10^4^	4.95 × 10^5^	6.65 × 10^5^	1.86 × 10^6^	5.01 × 10^7^	5.1 × 10^7^
740 kBq/kg (n = 3)	7.3 × 10^4^	1.29 × 10^6^	4.9 × 10^5^	2.6 × 10^6^	8.6 × 10^7^	7.07 × 10^5^
^225^Ac-DTPA (740 kBq/kg) (n = 2)	1.64 × 10^5^	4.3 × 10^5^	excluded	1.25 × 10^6^	3.9 × 10^6^	excluded

After seven days of follow-up, both implant and homogenized bone were sonicated for 10 min. Tenfold serial dilutions were cultured on Colombia Blood Agar plates in duplicate. CFUs were counted after overnight incubation in a 37 °C stove. Note: Due to continuous biting of the surgical wound, rat #3 of the ^225^Ac-DTPA (740 kBq/kg) treatment group was terminated after 72 h post-injection.

**Table A2:** Lutetium-177 treatment groups (177Lu-4497 and 177Lu-DTPA). The individual mean Colony Forming units (CFU) after seven days of follow-up.

	Implant (CFU)			Bone (CFU)		
	Rat #1	Rat #2	Rat #3	Rat #1	Rat #2	Rat #3
0 kBq/kg (n = 3)	1.7 × 10^6^	8.2 × 10^5^	1.95 × 10^6^	7.0 × 10^6^	3.4 × 10^8^	6.5 × 10^6^
112 MBq/kg (n = 3)	6.15 × 10^5^	7.3 × 10^5^	3.15 × 10^5^	6.2 × 10^7^	3.8 × 10^7^	9.6 × 10^7^
184 MBq/kg (n = 3)	1.5 × 10^5^	4.45 × 10^5^	5.3 × 10^5^	6.7 × 10^7^	1.47 × 10^5^	5.5 × 10^7^
260 MBq/kg (n = 3)	4.9 × 10^5^	5.0 × 10^5^	6.45 × 10^5^	5.4 × 10^7^	1.07 × 10^7^	4.0 × 10^7^
^177^Lu-DTPA (260 MBq/kg) (n = 3)	5.4 × 10^5^	7.25 × 10^5^	3.4 × 10^5^	5.4 × 10^8^	1.4 × 10^7^	6.3 × 10^6^

After seven days of follow-up, both implant and homogenized bone were sonicated for 10 min. Tenfold serial dilutions were cultured on Colombia Blood Agar plates in duplicate. CFUs were counted after overnight incubation 37 °C stove.

## References

[1] Nandakumar V., Chittaranjan S., Kurian V.M., Doble M. (2013). Characteristics of Bacterial Biofilm Associated with Implant Material in Clinical Practice. Polym. J..

[2] Ul Haq I., Khan T.A., Krukiewicz K. (2024). Etiology, Pathology, and Host-Impaired Immunity in Medical Implant-Associated Infections. J. Infect. Public Health.

[3] Van Epps J.S., Younger J.G. (2016). Implantable Device-Related Infection. Shock.

[4] Zhao A., Sun J., Liu Y. (2023). Understanding Bacterial Biofilms: From Definition to Treatment Strategies. Front. Cell. Infect. Microbiol..

[5] Arciola C.R., Campoccia D., Montanaro L. (2018). Implant Infections: Adhesion, Biofilm Formation and Immune Evasion. Nat. Rev. Microbiol..

[6] Seebach E., Kubatzky K.F. (2019). Chronic Implant-Related Bone Infections-Can Immune Modulation Be a Therapeutic Strategy?. Front. Immunol..

[7] Ciofu O., Moser C., Jensen P.Ø., Høiby N. (2022). Tolerance and Resistance of Microbial Biofilms. Nat. Rev. Microbiol..

[8] Sharma D., Misba L., Khan A.U. (2019). Antibiotics versus Biofilm: An Emerging Battleground in Microbial Communities. Antimicrob. Resist. Infect. Control.

[9] Michaelis C., Grohmann E. (2023). Horizontal Gene Transfer of Antibiotic Resistance Genes in Biofilms. Antibiotics.

[10] Rondon A., Rouanet J., Degoul F. (2021). Radioimmunotherapy in Oncology: Overview of the Last Decade Clinical Trials. Cancers.

[11] Brignoli T., Douglas E., Duggan S., Fagunloye O.G., Adhikari R., Aman M.J., Massey R.C. (2022). Wall Teichoic Acids Facilitate the Release of Toxins from the Surface of *Staphylococcus aureus*. Microbiol. Spectr..

[12] Brown S., Santa Maria J.P., Walker S. (2013). Wall Teichoic Acids of Gram-Positive Bacteria. Annu. Rev. Microbiol..

[13] de Vor L., van Dijk B., van Kessel K., Kavanaugh J.S., de Haas C., Aerts P.C., Viveen M.C., Boel E.C., Fluit A.C., Kwiecinski J.M. (2022). Human Monoclonal Antibodies against *Staphylococcus aureus* Surface Antigens Recognize In Vitro and In Vivo Biofilm. eLife.

[14] Ferdinandus J., Violet J., Sandhu S., Hofman M.S. (2018). Prostate-Specific Membrane Antigen Theranostics: Therapy with Lutetium-177. Curr. Opin. Urol..

[15] Yong K.J., Milenic D.E., Baidoo K.E., Brechbiel M.W. (2016). Mechanisms of Cell Killing Response from Low Linear Energy Transfer (Let) Radiation Originating from ^177^lu Radioimmunotherapy Targeting Disseminated Intraperitoneal Tumor Xenografts. Int. J. Mol. Sci..

[16] Sgouros G., Roeske J.C., McDevitt M.R., Palm S., Allen B.J., Fisher D.R., Brill A.B., Song H., Howell R.W., Akabani G. (2010). MIRD Pamphlet No. 22 (Abridged): Radiobiology and Dosimetry of α-Particle Emitters for Targeted Radionuclide Therapy. J. Nucl. Med..

[17] Danyo E.K., Ivantsova M.N., Selezneva I.S. (2024). Ionizing Radiation Effects on Microorganisms and Its Applications in the Food Industry. Foods Raw Mater..

[18] van Dijk B., Allen K.J.H., Helal M., Vogely H.C., Lam M.G.E.H., de Klerk J.M.H., Weinans H., van der Wal B.C.H., Dadachova E. (2020). Radioimmunotherapy of Methicillin-Resistant Staphylococcus Aureus in Planktonic State and Biofilms. PLoS ONE.

[19] Ye Z., van der Wildt B., Nurmohamed F.R.H.A., van Duyvenbode J.F.F.H., van Strijp J., Vogely H.C., Lam M.G.E.H., Dadachova E., Weinans H., van der Wal B.C.H. (2024). Radioimmunotherapy Combating Biofilm-Associated Infection In Vitro. Front. Med..

[20] Dadachova E., Howell R.W., Bryan R.A., Frenkel A., Nosanchuk J.D., Casadevall A. (2004). Susceptibility of the Human Pathogenic Fungi *Cryptococcus neoformans* and *Histoplasma capsulatum* to γ-Radiation Versus Radioimmunotherapy with α-and β-Emitting Radioisotopes. J. Nucl. Med..

[21] Dadachova E., Nakouzi A., Bryan R.A., Casadevall A. (2003). Ionizing Radiation Delivered by Specific Antibody Is Therapeutic against a Fungal Infection. Proc. Natl. Acad. Sci. USA.

[22] Eliopoulos G.M. (2012). E Therapy. Goldman’s Cecil Medicine.

[23] Pallares R.M., An D.D., Deblonde G.J.P., Kullgren B., Gauny S.S., Jarvis E.E., Abergel R.J. (2021). Efficient Discrimination of Transplutonium Actinides by in Vivomodels. Chem. Sci..

[24] Creff G., Safi S., Roques J., Michel H., Jeanson A., Solari P.L., Basset C., Simoni E., Vidaud C., Den Auwer C. (2016). Actinide(IV) Deposits on Bone: Potential Role of the Osteopontin-Thorium Complex. Inorg. Chem..

[25] Rubira L., Deshayes E., Santoro L., Kotzki P.O., Fersing C. (2023). ^225^Ac-Labeled Somatostatin Analogs in the Management of Neuroendocrine Tumors: From Radiochemistry to Clinic. Pharmaceutics.

[26] Wieszczycka K., Staszak K., Woźniak-Budych M.J., Jurga S. (2019). Lanthanides and Tissue Engineering Strategies for Bone Regeneration. Coord. Chem. Rev..

[27] Miederer M., Scheinberg D.A., McDevitt M.R. (2008). Realizing the Potential of the Actinium-225 Radionuclide Generator in Targeted Alpha Particle Therapy Applications. Adv. Drug Deliv. Rev..

[28] Davis I.A., Glowienka K.A., Boll R.A., Deal K.A., Brechbiel M.W., Stabin M., Bochsler P.N., Mirzadeh S., Kennel S.J. (1999). Comparison of ^225^Actinium Chelates: Tissue Distribution and Radiotoxicity. Nucl. Med. Biol..

[29] Vivier D., Sharma S.K., Zeglis B.M. (2018). Understanding the In Vivo Fate of Radioimmunoconjugates for Nuclear Imaging. J. Label. Comp. Radiopharm..

[30] Chérel M., Gouard S., Gaschet J., Saï-Maurel C., Bruchertseifer F., Morgenstern A., Bourgeois M., Gestin J.F., Bodéré F.K., Barbet J. (2013). ^213^Bi Radioimmunotherapy with an Anti-MCD138 Monoclonal Antibody in a Murine Model of Multiple Myeloma. J. Nucl. Med..

[31] Kristiansson A., Timmermand O.V., Altai M., Strand J., Strand S.E., Åkerström B., Örbom A. (2022). Hematological Toxicity in Mice After High Activity Injections of ^177^Lu-PSMA-617. Pharmaceutics.

[32] Di Bonaventura G., Pompilio A., Donelli G. (2022). In Vitro Antimicrobial Susceptibility Testing of Biofilm-Growing Bacteria: Current and Emerging Methods. Advances in Microbiology, Infectious Diseases and Public Health.

[33] Szafrańska A.K., Junker V., Steglich M., Nübel U. (2019). Rapid Cell Division of *Staphylococcus aureus* During Colonization of the Human Nose. BMC Genom..

[34] Larson S.M., Carrasquillo J.A., Cheung N.K.V., Press O.W. (2015). Radioimmunotherapy of Human Tumours. Nat. Rev. Cancer.

[35] Zhao J., Wen X., Li T., Shi S., Xiong C., Wang Y.A., Li C. (2020). Concurrent Injection of Unlabeled Antibodies Allows Positron Emission Tomography Imaging of Programmed Cell Death Ligand 1 Expression in an Orthotopic Pancreatic Tumor Model. ACS Omega.

[36] Van Der Veen E.L., Giesen D., Pot-De Jong L., Jorritsma-Smit A., De Vries E.G.E., Lub-De Hooge M.N. (2020). ^89^Zr-Pembrolizumab Biodistribution Is Influenced by PD-1-Mediated Uptake in Lymphoid Organs. J. Immunother. Cancer.

[37] Josefsson A., Nedrow J.R., Park S., Banerjee S.R., Rittenbach A., Jammes F., Tsui B., Sgouros G. (2016). Imaging, Biodistribution, and Dosimetry of Radionuclide-Labeled PD-L1 Antibody in an Immunocompetent Mouse Model of Breast Cancer. Cancer Res..

[38] du Sert N.P., Ahluwalia A., Alam S., Avey M.T., Baker M., Browne W.J., Clark A., Cuthill I.C., Dirnagl U., Emerson M. (2020). Reporting Animal Research: Explanation and Elaboration for the Arrive Guidelines 2.0. PLoS Biol..

[39] Allen K.J.H., Jiao R., Malo M.E., Frank C., Fisher D.R., Rickles D., Dadachova E. (2019). Comparative Radioimmunotherapy of Experimental Melanoma with Novel Humanized Antibody to Melanin Labeled with 213bismuth and 177lutetium. Pharmaceutics.

[40] Allen K.J.H., Frank C., Jiao R., Malo M.E., Bello M., De Nardo L., Meléndez-Alafort L., Dadachova E. (2024). In Vitro and In Vivo Comparison of Random Versus Site-Specific Conjugation of Bifunctional Chelating Agents to the CD33-Binding Antibody for Use in Alpha- and Beta-Radioimmunotherapy. ACS Omega.

[41] Boles B.R., Thoende M., Roth A.J., Horswill A.R. (2010). Identification of Genes Involved in Polysaccharide-Independent *Staphylococcus aureus* Biofilm Formation. PLoS ONE.

[42] Giknis M., Clifford C. (2008). Clinical Laboratory Parameters for CrL:WI (Han).

